# Enhanced virulence of *Histoplasma capsulatum* through transfer and surface incorporation of glycans from *Cryptococcus neoformans* during co-infection

**DOI:** 10.1038/srep21765

**Published:** 2016-02-24

**Authors:** Radames J. B. Cordero, Susie Coutinho Liedke, Glauber R. de S. Araújo, Luis R. Martinez, Leonardo Nimrichter, Susana Frases, Jose Mauro Peralta, Arturo Casadevall, Marcio L. Rodrigues, Joshua D. Nosanchuk, Allan J. Guimaraes

**Affiliations:** 1 Department of Molecular Microbiology and Immunology, Johns Hopkins Bloomberg School of Public Health, Baltimore, MD, US; 2 Departamento de Imunologia, Instituto de Microbiologia Professor Paulo de Góes, Universidade Federal do Rio de Janeiro, RJ, Brazil; 3 Laboratório de Ultraestrutura Celular Hertha Meyer, Instituto de Biofísica Carlos Chagas Filho, Universidade Federal do Rio de Janeiro & Instituto Nacional de Metrologia, Qualidade e Tecnologia (Inmetro), Rio de Janeiro, Brazil; 4 Department of Biomedical Sciences, College of Osteopathic Medicine; New York Institute of Technology; Old Westbury, NY, US; 5 Laboratório de Glicobiologia de Eucariotos, Instituto de Microbiologia Paulo de Góes, Universidade Federal do Rio de Janeiro, RJ, Brazil; 6 Laboratório de Biologia Celular de Leveduras Patogênicas, Instituto de Microbiologia Professor Paulo de Góes, Universidade Federal do Rio de Janeiro, RJ. Brazil; 7 Fundação Oswaldo Cruz – Fiocruz, Centro de Desenvolvimento Tecnológico em Saúde (CDTS), Rio de Janeiro, Brazil; 8 Department of Microbiology and Immunology, Albert Einstein College of Medicine of Yeshiva University, Bronx, NY, US; 9 Department of Medicine (Division of Infectious Diseases), Albert Einstein College of Medicine of Yeshiva University, Bronx, NY, US; 10 Departamento de Microbiologia e Parasitologia, Instituto Biomédico, Universidade Federal Fluminense, Rio de Janeiro, Brazil

## Abstract

*Cryptococcus neoformans* (*Cn*) and *Histoplasma capsulatum* (*Hc*) co-exist in the environment and occasionally co-infect individuals, which can lead to severe disease/lethal outcomes. We investigated specific interactions between *Cn*-*Hc* to determine the impact of synchronous infection in virulence and disease. Co-infected mice had significantly higher mortality than infection with either species or acapsular *Cn*-*Hc*. Coating of *Hc* with cryptococcal glycans (*Cn*-gly) resulted in higher pulmonary fungal burden in co-infected animals relative to control. Co-cultivation or addition of *Cn*-gly resulted in enhanced pellicle formation with a hybrid polysaccharide matrix with higher reactivity to GXM mAbs. Transfer and incorporation of *Cn* polysaccharide onto *Hc* surface was time and temperature dependent. *Cn-*gly transfer altered the zeta potential of *Hc* and was associated with increased resistance to phagocytosis and killing by macrophages. Mice infected with *Hc* and subsequently injected with purified *Cn*-gly died significantly more rapidly than *Hc* alone infected, establishing the precedent that virulence factors from one fungus can enhance the virulence of unrelated species. These findings suggest a new mechanism of microbial interaction involving the transfer of virulence traits that translates into enhanced lethality during mixed fungal infections and highlights the importance of studying heterogeneous microbial populations in the setting of infection.

*Cryptococcus neoformans* (*Cn*) and *Histoplasma capsulatum* (*Hc*) are two of the major pathogenic fungi in the world, causing millions of infections annually with significant morbidity and mortality^1^,^2^. *Hc* is a dimorphic fungus responsible for a wide range of clinical presentations, from asymptomatic infection or a mild influenza-like illness to disseminated sepsis^3^ that is frequently associated with fatal infection. Epidemiological studies have estimated that ~500,000 individuals acquire *Hc* annually in the USA and over 80% of young adults in endemic areas have been infected by the fungus^4^,^5^. Fortunately, the majority of individuals acquiring *Hc* do not develop clinically significant infections, although there are still ~3,500 hospitalizations due to histoplasmosis in the USA annually, with a crude mortality rate of ~8%^5^. Pulmonary histoplasmosis symptoms are similar to those of pulmonary cryptococcosis, caused by the encapsulated basidomycetous yeast *Cn* and/or *C. gattii*. In addition, cryptococcosis can evolve to a life-threatening meningoencephalitis in susceptible individuals^1^. Globally, cryptococcal meningitis occurs in about 1 million individuals annually with a mortality rate of approximately 60%^1^. Individuals with histoplasmosis or cryptococcosis who are iatrogenically immunosuppresed (ie. receiving steroids or tumor necrosis factor-alpha inhibitors) or have impaired cell-mediated immunity (ie. HIV patients) are at high risk for life-threatening disease^6^.

*Hc* and *Cn* are widely distributed in the environment and infection by either/both fungi can be acquired after disturbance and aerosolization of soil contaminated with bird excreta^1^. Although co-infection rates are unknown, most adults in urban areas have serological evidence of *Cn* infection^7^ and skin testing has shown a high prevalence of *Hc* infection in endemic areas^8^. Consequently, it is possible that in *Hc* endemic areas there are high numbers of individuals who have been infected with both *Hc* and *Cn,* although there is no information on the timing of these infections (ie. acquisition occurring concomitantly or separately). Nevertheless, a review of the literature finds a significant number of cases of *Cn-Hc* co-infections^9^,^10^,^11^,^12^,^13^,^14^,^15^,^16^,^17^,^18^,^19^,^20^,^21^, which establishes that co-infections can and do occur, and can progress to disease with both fungi. Identification of co-infected individuals is complicated by the fact that clinical manifestations of both mycoses and the antifungal therapy administered for them are similar (typically a polyene followed by an azole). Additionally, *Cn* is more likely to be identified by routinely microscopy techniques and grows within 5 days on Sabouraud agar, whereas *Hc* is more fastidious, typically takes about 14-to-30 days for growth in culture^1^,^8^ and can also be inhibited by *Cn*^22^. Hence, it is probable that co-infection is under diagnosed and that the true incidence of concomitant infection is significantly greater than currently understood.

Many components of the cell wall of *Cn* are similar to those of *Hc,* and these surface components form the main interacting interface with their environment and cells of the host immune system. However, the outer layer of *Cn* consists of an additional large anti-phagocytic polysaccharide (PS) capsule, which is the fungus’ most distinctive virulence determinant. The capsule is mainly composed of glucuronoxylomannan (GXM), a high molecular mass (10^6^–10^8^ g/mol) α-1, 3-linked mannan backbone decorated with xylose and glucuronic acid residues^23^. GXM is synthesized intracellularly within the Golgi and released via vesicles to the extracellular milieu^24^. Eventually the GXM is incorporated into a growing capsule by complex PS-PS interactions that include GXM interaction with cell wall-derived α-glucans^25^, chitin-derived structures^26^, and other GXM molecules^27^. Significantly, GXM is also released into the serum and tissues during disease, frequently reaching titers >1:10,000 (or >10 μg/mL) in human disease^28^; hence, there is ample opportunity for the PS to interact *in vivo* with other microbes as well as host cells. In fact, in addition to protecting the fungus against oxidative stress^23^, *Cn* capsular PS is associated with potent detrimental effects on the immune system, such as inhibition of phagocytosis, dysregulation of immunoresponses, reduced leukocyte migration, complement depletion, interference in antigen presentation, and T-cell suppression with subsequent inhibition of inflammatory cytokines production^29^. Additional roles of the cryptococcal capsule in virulence have been demonstrated using congenic strain pairs that differ only by mutations or replacement of specific capsular synthesis/assembly genes, such as the well characterized CAP genes family (CAP10, CAP59, CAP60, CAP64), CAS genes (CAS1, CAS3, CAS31) and many others^23^,^30^. These mutations result in acapsular or hypocapsular phenotypes^23^ that were severely attenuated in murine models of infection^30^.

The outer layer of *Hc* yeast cells displays several surface molecules involved in their internalization by various phagocytes and several carbohydrate-linked structures with immunomodulatory activities are intimately linked to fungal pathogenesis and virulence^31^. Known *Hc* glycans (gly) include chitin, α-1, 3- and β-1, 3-glucans, and extracellular galactomannan^32^. Although the *Hc* surface is less well understood relative to that of *Cn,* and only a few gly have been partially characterized, *Hc* can incorporate exogenously added cryptococcal exoPS *in vitro*^25^. However, it is unclear whether PS transfer occurs in the environment or during mammalian co-infection. Moreover, the importance of this process on the outcome of *Hc* infection has not been explored.

In this study, we explored whether co-infection with *Cn* affected the virulence of *Hc*. Incorporation and coating of *Hc* yeast cells by *Cn* PS was detected during co-infection of mice. This incorporation of *Cn* PS by *Hc* increased pulmonary disease, as there were higher fungal burdens of encapsulated *Hc* in the lungs of co-infected mice compared to mice infected with *Hc* alone. The acquisition and incorporation of exogenous GXM on *Hc* yeast cell surfaces altered the cellular electrostatic potential and resulted in a reduction in phagocytosis and intracellular killing of the yeast by macrophages. The observations presented in this work raise the possibility that fungi can interchange virulence factors and that this process can modulate the immune response and lead to enhanced damage to mammalian hosts.

## Results

### *Co*-infection resulted in enhanced mouse mortality

**Figure 1:**
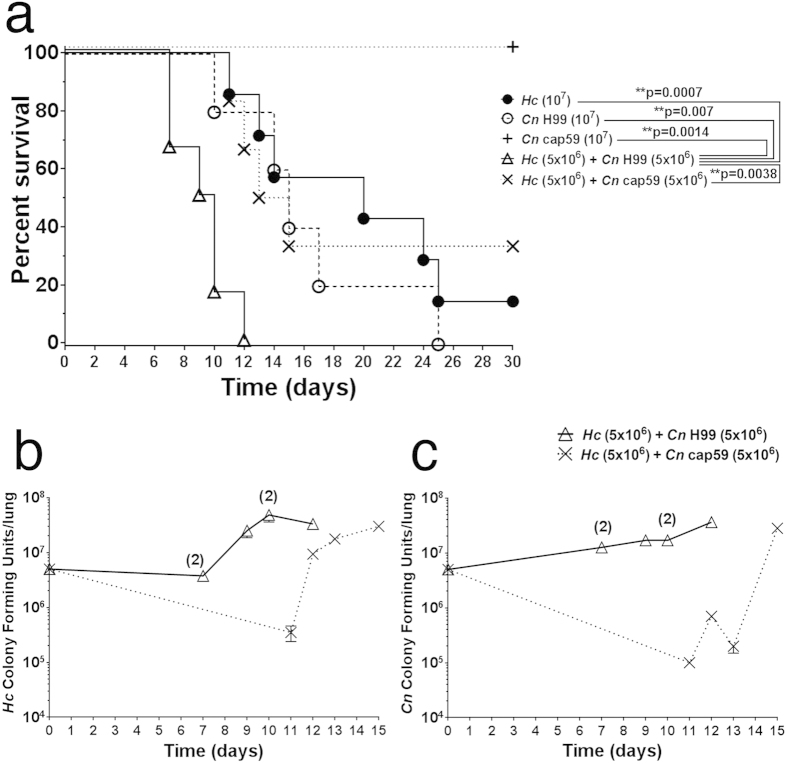
Co-infection of mice with *Hc* and encapsulated *Cn* enhances virulence. (**a**) Mice were infected with 10^7^ total yeast inoculated as either a single species (10^7^ of either *Cn* H99 or *Hc*) or a 1:1 mix of both fungi (5 × 10^6^ *Hc* with 5 × 10^6^ of either *Cn* H99 or *Cn* cap59). Co-infection with *Hc* and *Cn* H99 resulted in accelerated mortality compared to the other groups. (**b**) *Hc* and (**c**) *Cn* pulmonary CFUs from animals who expired due to infection with *Hc* and either *Cn* H99 or *Cn* cap59. Time 0 indicates initial inoculum of the specified fungi. Pulmonary fungal burdens of both. (**b**) *Hc* and (**c**) *Cn* were relatively higher for mice infected with *Hc* + *Cn* H99 compared to *Hc* co-infected with the acapsular *Cn* cap59. When present, digits over graph points in b and c indicate the number of deceased animals at a specific time point (cumulative death/same day), with CFUs expressed as averages.

We explored the possibility that *Cn* and *Hc* co-infection could worsen disease prognosis in mice. Co-infection was assessed with an equal mixed inoculum of *Hc* and encapsulated *Cn* H99 or unencapsulated *Cn* cap59 (5 × 10^6^ of each fungus) and compared with monospecies infected animals (10^7^ yeasts). Co-infection with *Hc* and *Cn* H99 resulted in higher mortality rates, with 100% death after 12 days, relative to mice infected with *Hc* and acapsular *Cn* cap59 (p = 0.0038) or monospecies inoculum of either *Hc* (**p = 0.0007), *Cn* H99 (**p = 0.007) or *Cn* cap59 (**p = 0.0014; Fig. 1a).

To confirm that animals were indeed co-infected and to determine fungal burdens in the scope of *Cn* PS importance, the colony forming units (CFU) were determined in lungs of deceased animals in the course of the survival experiments for *Hc* (Fig. 1b) and *Cn* (Fig. 1c). Both fungal species were recovered in similar proportions, indicating they colonize the lungs with similar efficacy and that they could interact *in vivo*.

However, animals from the *Hc* + *Cn* H99 co-infection group had higher burdens of *Hc* and *Cn* during earlier time-points than those in the *Hc* + *Cn* cap59 group (Fig. 1b,c, respectively), which correlated with the increased lethality of the *Hc* co-infection with the encapsulated *Cn*. *Hc* fungal burdens from *Hc* + *Cn* H99 group varied from 2.9 × 10^6^ to 7.0 × 10^7^ (median 2.6 × 10^7^), while the *Hc* + *Cn* cap59 group ranged from 3.5 × 10^5^ to 3.0 × 10^7^ (median 1.35 × 10^7^). For *Cn* fungal burdens, group *Hc* + *Cn* H99 ranged from 1.2 × 10^7^ to 3.6 × 10^7^ (median 1.7 × 10^7^), while *Hc* + *Cn* cap59 group ranged from 1.0 × 10^5^ to 2.8 × 10^7^ (median 4.5 × 10^5^). These results suggest that co-infection of *Hc* with *Cn* that efficiently releases PS leads to an increase in the virulence of *Hc in vivo*. Additionally, the monoinfection with *Hc* yeast cell alone resulted in fungal burdens that were ~25% lower than the average observed for the *Hc* + *Cn* H99 co-infected animals, further suggesting that virulence of *Hc* is enhanced in the presence of *Cn*. This conclusion is further justified based on the fact that half as many *Hc* yeast cells (5 × 10^6^) were introduced to the co-infected animals compared to those receiving *Hc* alone (10^7^).

### *Hc* incorporates *Cn* glycans *in vivo*

**Figure 2:**
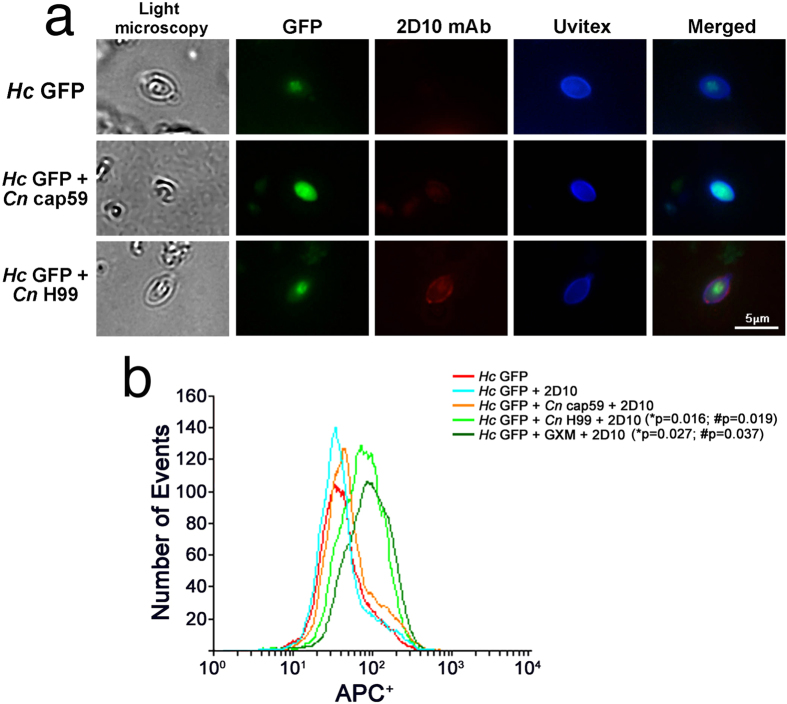
*Hc* incorporates *Cn*-glycans *in vivo* during co-infection. (**a**) *Hc* binds *Cn-*gly during co-infection with *Cn* H99. Immunofluorescence punctuate surface labelling of *Hc* GFP recovered from lungs of *Hc* GFP+*Cn* H99 groups with GXM-binding mAb 2D10 (red) and Uvitex2B (chitin in the cell wall) after isolation from lungs of co-infected animals. In comparison, *Hc* recovered from lungs of *Hc* GFP+*Cn* cap59 co-infected or monospecies (*Hc* GFP) infected mice are not labelled by the mAb. Scale bar = 5 μm. (**b**) FACS demonstrates labelling of *Hc* cells by GXM 2D10 mAbs upon co-infection with *Cn* H99 or GXM added controls (in comparison to unlabelled *Hc* GFP (*p = 0.013 and **p = 0.0069, respectively) and *Hc* yeast from co-infection with *Cn* cap59 (^#^p = 0.037 and ^#^p = 0.019).

The possibility that *Hc* could interact *in vivo* with *Cn-*glycans was explored initially by the evaluation of the transfer of *Cn* PS to the surface of *Hc*. Recovered yeasts from co-infected lungs were incubated with 2D10 mAbs to cryptococcal GXM^33^ and an anti-mouse IgM Alexa 546 conjugate and evaluated by fluorescence microscopy (Fig. 2a). *Hc* GFP yeasts recovered from *Hc* + *Cn* H99 co-infected lungs displayed strong labelling for GXM in comparison to the absence of labelling on fungal cells obtained from mice infected with *Hc* + *Cn* cap59 or *Hc* GFP control alone (Fig. 2a). To obtain more quantitative information, *Hc* yeasts recovered from infected mice were incubated with mAb 2D10 and anti-mouse IgM allophycocyanin (APC)-conjugate and evaluated by flow cytometry (Fig. 2b). As an additional control, GXM was exogenously added to *Hc* samples. As expected, *Hc* from *Hc* + *Cn* cap59 co-infected animals displayed low levels (background) of APC^+^, similar to *Hc* incubated with 2D10 mAb (p = 0.44) or *Hc* alone (p = 0.52) and suggested no labelling by 2D10 mAbs. *Hc* isolated from lungs of *Hc* + *Cn* H99 co-infected animals had higher fluorescence labelling by mAbs to GXM than to *Hc*+2D10 (*p = 0.016) or *Hc* + *Cn* cap59 (^#^p = 0.019), indicating incorporation *in vivo*. Notably, the APC intensity values were similar for the *Hc* yeast isolated from animals co-infected with the PS-producing *Cn* H99 and isolated *Hc* spiked with GXM (p = 0.23). These results suggest that during co-infections the surface of Hc can be modified by the incorporation of cryptococcal PS material.

### *Cn* and *Hc* interacted during co-cultivation

**Figure 3:**
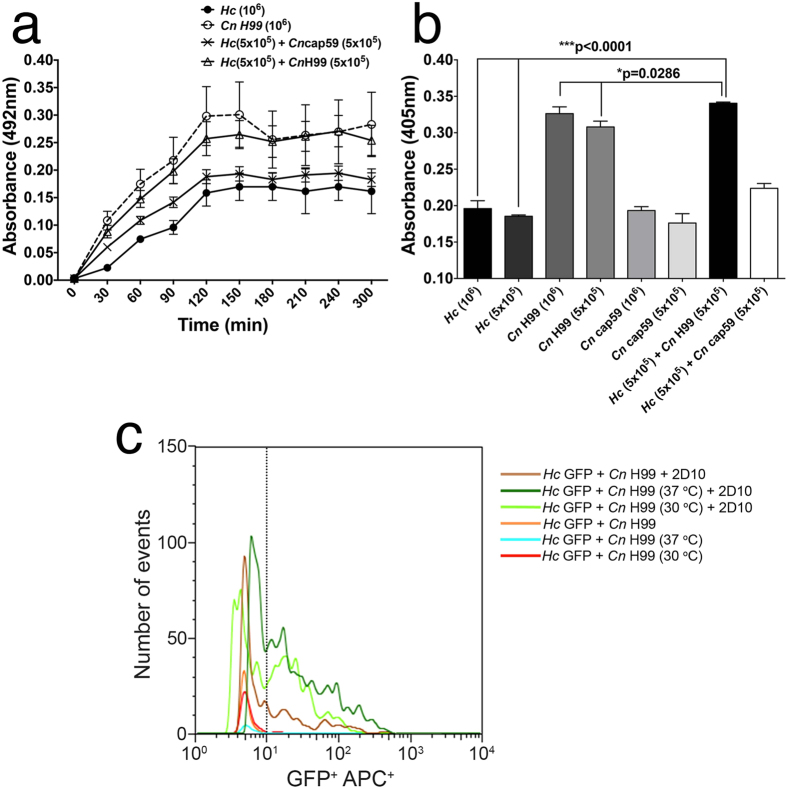
Co-cultivation of *Cn* H99 and *Hc* GFP enhances pellicle formation by glycan transfer. (**a**) Pellicle formation in HAM’s F-12 media was determined by measuring fungal metabolic activity using XTT colorimetric analysis. The initial inoculum for each well was 10^6^ yeast cells, either all of one species or a 1:1 mix of 5 × 10^5^ of *Hc* co-cultured with either *Cn* H99 or *Cn* cap59. Heat-killed *Hc* cells were used as background control and discounted from the readings. Co-cultures of *Hc* GFP + *Cn* H99 formed pellicles that were similar to biofilms produced by monospecies *Cn* H99. In contrast, *Hc* GFP + *Cn* cap59 and monospecies *Hc* GFP were extremely poor pellicle producers. (**b**) The reactivities of PS matrix of fungal pellicles were examined by ELISA using 2D10 IgM mAb to GXM. The pellicles from *Hc* GFP + *Cn* H99 displayed a slight, but significant increase in matrix reactivity to GXM-binding mAb compared to biofilms formed by *Cn* H99 alone. For both A and B, bars represent mean ± standard error of quadruplicates. (**c**) *Cn* glycan transfer to *Hc* surface is temperature dependent. More glycan transfer occurs at 37 °C compared to 30 °C during co-cultivation of *Hc* (GFP strain, FL1-H^+^) +*Cn* H99 in HAM’s F-12 medium as determined by flow cytometry using GXM-binding mAb 2D10 (IgGM) and a goat anti-mouse IgM-APC (FL4-H^+^), in comparison to controls or monospecies mixed *Hc* GFP + *Cn* H99 yeasts just before incubations with mAb, which displayed no PS transfer.

Given that both fungi can co-exist in nature and in tissues, we evaluated the interactions between *Cn* and *Hc* during *in vitro* cultivation. Fungal growth was examined semi-quantitatively on microtiter plates by measuring total metabolic activity of adherent cells and pellicle formation using an XTT assay (Fig. 3a)^34^. When cultivated separately, *Cn* H99 grows more robustly under biofilm conditions compared to *Hc* (*p = 0.016, 2 h), which is consistent with the differences in replication rate between the two fungi (approximately 2 and 6 h, respectively) and the well-described capacity of *Cn* to form a biofilm/PS matrix^23^,^34^,^35^. However, co-incubation of both fungi in a 1:1 ratio to create the same total initial inoculum, resulted in the formation of a hybrid pellicle, with similar metabolic activity relative to monospecies *Cn* biofilms (p = 0.46). The capacity of forming pellicles was nearly absent in *Hc* + *Cn* cap59 co-cultivation, where the metabolic activity was 27% lower relative to pellicles containing *Hc* and *Cn* H99 (*p = 0.029, 2h). 3-D image reconstruction of pellicles displayed the complex architecture formed when *Hc* was co-cultured in static conditions with *Cn* H99, in comparison with monospecies control and *Hc* + acapsular *Cn* cap59 and (Supplementary Fig. S1a–c) along with the detection of fluorescence intensity (Supplementary Fig. S1f), and correlated with the above described results.

To examine the PS matrix of these fungal pellicles, we performed an indirect ELISA using mAb 2D10 to cryptococcal GXM. Mixed *Hc* + *Cn* H99 pellicles displayed an average reactivity increase of 10% relative to *Cn* biofilms (Fig. 3b, *p = 0.029). No difference on the reactivity was observed when comparing mixed pellicles of *Hc* + *Cn* cap59 with pellicles from *Hc* yeasts alone (p > 0.99). These results also suggest that PS material from *Cn* is transferred to *Hc* and that these fungi form a hybrid pellicle matrix with increased serological reactivity. Altogether, these findings might suggest an interplay between *Cn* and *Hc* when grown together. In fact, *Cn* also has been reported to produce quorum sensing molecules that affect the growth of other fungi^36^.

### Temperature dependency of cryptococcal glycan incorporation by *Hc*

*Hc* cells were individually evaluated by FACS upon incubation with mAb 2D10 and APC-labelled conjugate anti-mouse IgM after sonication of grown *Hc* + *Cn* H99 co-cultures at 30 °C and 37 °C. The optimal growth temperature for *Cn* is 30 °C and *Hc* yeast cells grown best at 37 °C. Co-cultivation of *Hc* (GFP) and *Cn* H99 resulted in transfer and incorporation of *Cn-*glycan fractions on the *Hc* surface (Fig. 3c), related to mixed monospecies control (*Hc* + *Cn* H99), which barely had increase in *Hc* fluorescence in comparison to *Hc* control. Incubation of cells at 37 °C resulted in a 5-fold increase in average APC^+^ fluorescence intensity of GXM-positively labeled *Hc* compared to *Hc* grown at 37 °C, while cells co-cultured at 30 °C displayed a 3-fold increase in average fluorescence compared to *Hc* grown at the same temperature.

### Cellular glycan cross-incorporation between *Hc* and *Cn*

Based on the incorporation of *Cn* PS by *Hc*, we investigated also whether both thermodimorphic phases of *Hc* would incorporate *Cn*-glycan, since these fungi co-exist in nature in a wide variety of temperatures. When evaluated by fluorescence microscopy, the majority of *Hc* yeast cells in pure culture were not labelled by mAb 18B7^37^ although few cells displayed a discrete punctuated pattern of labelling (Supplementary Fig. S2a). The filamentous phase of *Hc* also displayed only few cells labelled by mAb 18B7 some concentrated at the septae (Supplementary Fig. S2d). To evaluate the *Cn*-glycan incorporation, as previously described for GXM and *Hc*^25^, *Hc* yeasts were incubated with *Cn*-glycan resulting in a radial labelling surrounding the *Hc* surface or “pseudoencapsulation” of *Hc* by the *Cn*-glycan (Supplementary Fig. S2b). The surface of filamentous forms was also able to incorporate *Cn-*glycan onto it’s the surface, with the most intense fluorescence surrounding micro and macroconidia (Supplementary Fig. S2e). Pre-treatment of yeast or hyphal cells with the cell wall degrading cocktail Novozyme completely abrogated the binding of *Cn-*glycan (Supplementary Fig. S2c,f), suggesting the requirement of cell surface molecules in this process.

### Incorporation of distinct cryptococcal glycan fractions onto the *Hc* surface

**Figure 4:**
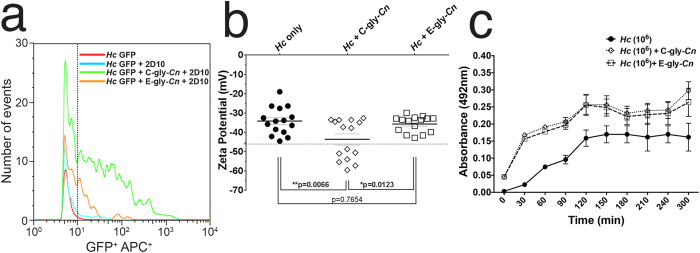
*Hc* incorporates distinct *Cn-*gly fractions on its surface. (**a**) Flow cytometry of GFP + *Hc* yeast (FL1-H^+^) following incubation with purified C-gly-*Cn* and E-gly-Cn and 2D10-APC conjugate reveals that C-gly-*Cn* is incorporated more effectively onto the cell surface by *Hc* compared to E-gly-*Cn*. (**b**) *Cn*-glycan surface incorporation changes *Hc* surface charge. Dashed error bars represent the standard error of average zeta potential values obtained from 10 repeated measurements. (**c**) Incorporation of C-gly-*Cn* or E-gly-*Cn* by *Hc* enabled the formation of pellicles by the fungus.

The attachment or anchoring of the *Cn* capsule involves PS-PS interactions between GXM and other cell wall gly (i.e. glucans and chitin)^25^,^26^. Given that the *Hc* surface is richly composed of glucans and N-acetylglucosamine polymers^32^, we examined the possibility of a transference and/or incorporation of distinct cryptococcal PS fractions onto the *Hc* surface. This was assessed by incubation of *Hc* cells with isolated capsular C-gly-*Cn* (DMSO extracted) and secreted E-gly-*Cn* (filtered supernatant) fractions, isolated from *Cn*. The C-gly-*Cn* fraction was readily incorporated by *Hc* yeasts based on a 7-fold increase in mAb 2D10 labelling relative to control (Fig. 4a). The E-gly-*Cn* fraction was less well incorporated; nevertheless, incubation with this fraction led in a 4-fold increase in antibody labelling.

C-gly-*Cn* incorporation modified the charge of *Hc* as demonstrated by the change in the surface electrostatic potential of *Hc* cells. The association of C-gly-*Cn* with *Hc* cells resulted in a significant increase in the negative magnitude of the zeta potential (−46.56 ± 10.25 mV) relative to uncoated *Hc* yeasts (−34.10 ± 7.10 mV, *p = 0.0066) (Fig. 4b), most likely due to the addition of glucuronic acid residues, which are absent on *Hc* surface gly. Incubation with E-gly-*Cn* not significantly alter *Hc* surface charge (−35.63 ± 4.18 mV), consistent with the lower incorporation and the lower relative levels of glucuronic acid in this fraction compared to C-gly-*Cn*^38^. Together, these results demonstrate an interaction between both fungi involving the transfer and incorporation of *Cn* PS material to the *Hc* surface gly via PS-PS interactions, which leads to significant alterations in *Hc* cell surface charge.

Growth of *Hc* in the presence of C-gly-*Cn* or E-gly-*Cn* enabled these yeasts to more effectively form a pellicle structure (Fig. 4c) equally in the presence of either glycan fraction (p = 0.77, 2 h), relative to *Hc* alone (*p = 0.037 and p = 0.045, respectively). Structurally, these pellicles were characterized by dense aggregates of yeasts, which could anchor each other through interactions with *Cn*-gly working as a extracellular polymeric scaffold substance (Supplementary Fig. S1d,e).

**Figure 5:**
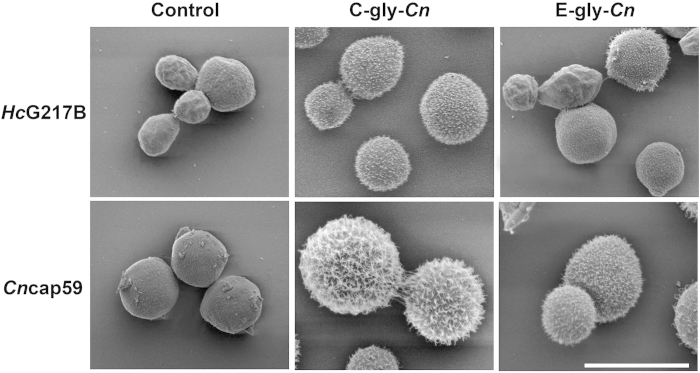
Incorporation of cryptococcal glycan fractions by *Hc* or acapsular *Cn* cap59 mutant produces distinct cell surface architectural features. Scanning electron microscopy (SEM) images of *Hc* and acapsular cap59 *Cn* mutants display distinct arrangements of C-gly-*Cn* and E-gly-*Cn* on their surfaces. Both yeast species produced more complex structures through the incorporation of C-gly-*Cn* in comparison E-gly-*Cn*. Scale bar = 5 μm.

The post-incorporation ultrastructure of *Hc* was evaluated by SEM (Fig. 5). As a control, *Cn* cap59 yeasts were incubated with C-gly-*Cn* or E-gly-*Cn* and uniform attachment of capsule was observed, with C-gly-*Cn* producing the most robust capsules, in comparison to E-gly-*Cn*, and agreement with the previously described size of PS fibers from these distinct fractions^38^. Similarly, *Hc* yeasts incubated with C-gly-*Cn* displayed significantly larger PS fibers incorporated onto their surface compared to the smoother surface by E-gly-*Cn*, which had a more sparsely coated surface, but more wrinkled than *Hc* control. Together, these resulted were consistent with the FACS and zeta-potential determinations.

### Kinetics of *Cn* glycan incorporation by *Hc* and α-glucan requirement

The requirement for α-1, 3-glucans in the incorporation of C-gly-*Cn* or E-gly-*Cn* was evaluated by comparing *Hc* strains expressing variable amounts of these surface glucans. Decreasing concentrations of C-gly-*Cn* or E-gly-*Cn* were incubated up to 1 h with low (G217B) or high α-1, 3-glucan content (G186A) *Hc* strains^39^. C-gly-*Cn* was incorporated by both *Hc* strains (Supplementary Fig. 3Sa,b). Despite higher incorporation of C-gly-*Cn* by G186A, in agreement with Reese *et al.*^25^, this process was effective only at 1 h incubation, in contrast with G217B strain, which displayed a statistically significant incorporation of C-gly-*Cn* after a 30 min incubation, when compared to controls. Similar behaviour was observed for both strains with E-gly-*Cn* incubation; however, as expected, absorbance values were lower than those obtained for C-gly-*Cn,* due to the lower incorporation of this fraction by *Hc* strains.

### *Cn* glycans-coated *Hc* yeasts are more resistant to phagocytosis and antifungal activity by peritoneal macrophages

**Figure 6:**
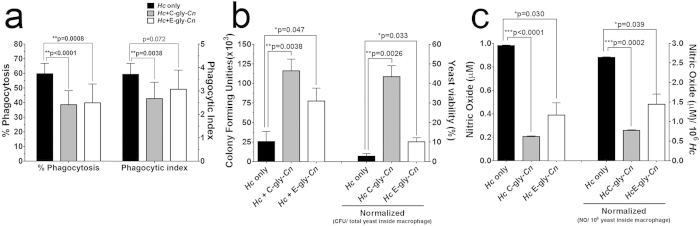
Cn-gly surface incorporation by *Hc* affects subsequent interactions with macrophages. The incorporation of C-gly-*Cn* or E-gly-*Cn* onto the surface of *Hc* significantly increased its resistance to (**a**) phagocytosis and (**b**) killing by murine peritoneal macrophages. Bars represent mean ± standard error of quadruplicates. (**c**) Co-culture of macrophages with *Hc* cells coated with *Cn*-gly suppressed the production of nitric oxide by macrophages. Bars represent mean ± standard error of three independent experiments performed in triplicates.

Given the antiphagocytic and immunomodulatory properties of cryptococcal PS, we examined if these virulence traits could occur with pseudoencapsulated *Hc* cells. *Hc* yeasts coated with C-gly-*Cn* were more resistant to phagocytosis by peritoneal macrophages compared to untreated *Hc* (38.6% vs 59.8% phagocytosed, ***p = 0.0001; Fig. 6a). Similar results were achieved with E-gly-*Cn* incorporation onto *Hc* (39.5%, **p = 0.0008). When the phagocytosis index was evaluated, i.e., the average number of yeast by macrophages, only C-gly-*Cn* reduced this number effectively (2, 67; **p = 0.0013), in comparison to E-gly-*Cn* (3,07; p = 0.072) and *Hc* control (3, 71). Moreover, resistance to killing by macrophages was also increased for *Hc* coated with C-gly-*Cn*, as the CFUs were 4.3 times higher for these cells compared to uncoated *Hc* (1.2 × 10^5^ vs colonies 2.6 × 10^4^, **p = 0.0038; Fig. 6b, left axis). E-gly-*Cn* coated-*Hc* similarly displayed a 3 times higher resistance to intracellular killing (7.8 × 10^4^, *p = 0.047) compared to untreated *Hc* (2.6 × 10^4^). Resistance to killing (CFU) was normalized by the total yeast number inside the macrophages and yeast viability under each condition evaluated (Fig. 6b, right axis). This reduced macrophage antimicrobial efficacy was in part associated with the decreased levels of nitric oxide produced by these effector phagocytic cells when infected with C-gly-*Cn* or E-gly-*Cn* coated *Hc* (p < 0.05; Fig. 6c, left axis). Nitric oxide levels were normalized to the number of yeast inside macrophages (p < 0.05; Fig. 6c, right axis). These results suggest that interaction between *Cn* and *Hc* can result in the generation of *Hc* cells with a cryptococcal-like surface and, thus, new and/or hybrid virulence properties, including ability to grow more efficiently within phagocytes and inhibition of nitric oxide production.

### *Hc* virulence is enhanced *in vivo* via *Cn* glycan transfer

**Figure 7:**
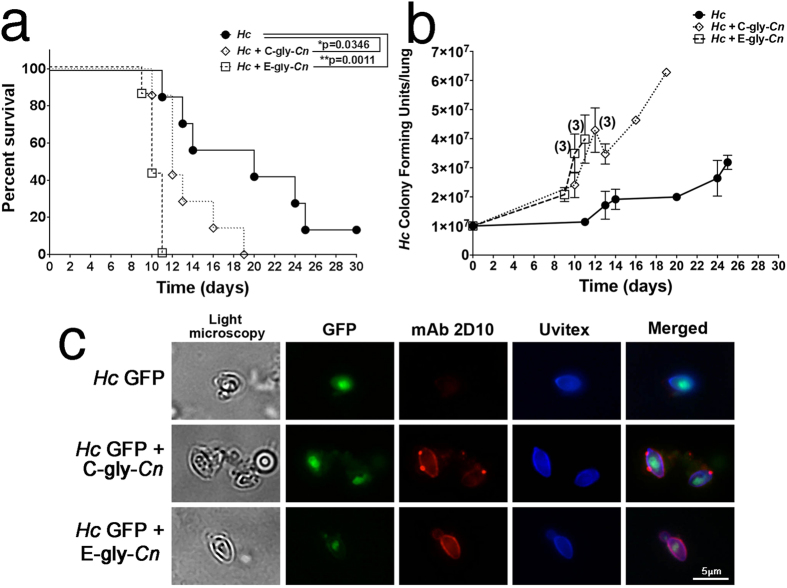
Virulence is enhanced by the incorporation of cryptococcal glycan fractions onto the surface of *Hc* yeast in a murine infection model. (**a**) Enhanced mortality occurred when mice were infected with *Hc* cells and subsequently injected with E-gly-*Cn* or C-gly-*Cn* in comparison with Hc infected animals. (**b**) Mice treated with C-gly-*Cn* or E-gly-*Cn* after infection with *Hc* displayed higher fungal burdens in comparison to animals infected with *Hc* alone. When present, digits over graph points reveal the number of deceased animals at a specific time point, with CFUs expressed as averages. Results are representative of two-independent experiments with 7 animals per group. (**c**) *Hc* binds C-gly*-Cn* or E-gly-*Cn in vivo,* displaying a punctuate surface labelling of *Hc* GFP recovered from lungs of mice administered with the distinct pool of *Cn*-gly by GXM-binding mAb 2D10 (red) and Uvitex2B. In comparison, *Hc* recovered from lungs of monospecies infected *Hc* mice are not labelled by the mAb. Scale bar = 5 μm.

*In vivo* mouse models were used to determine the importance of the transfer of individual pools of *Cn*-gly during co-infection *in vivo*. After infection with *Hc* and administration of C-gly-*Cn* or E-gly-*Cn* intratracheally, survival rates and lung CFUs were compared. Animals challenged with *Hc* and treated with E-gly-*Cn* had the highest mortality index, with all mice dying by day 11 (**p = 0.0011 compared to *Hc* infection alone, Fig. 7a), followed by mice in the C-gly-*Cn* treatment group, with animals dying by day 19 (*p = 0.035). Notably, some animals infected with *Hc* that received PBS instead of PS survived until the termination of the experiment at day 30. The CFUs recovered from *Hc-*infected and C-gly-*Cn* treated animals ranged from 1.98 × 10^7^ to 6.28 × 10^7^ (median 4.64 × 10^7^) and the CFUs from infected, E-gly-*Cn* treated mice ranged from 1.71 × 10^7^ to 6.23 × 10^7^ (median 2.71 × 10^7^), both of which were significantly higher than CFUs recovered from mice infected with *Hc* alone (1.14 × 10^7^ to 3.19 × 10^7^; median 1.95 × 10^7^; p < 0.05; Fig. 7b). Since CFU numbers were higher in animals treated with either C-gly-*Cn* or E-gly-*Cn* than control, we wanted to determine if the higher virulence was correlated with the presence of *Hc*-coated yeasts with the administered *Cn*-gly. Significantly, organ homogenates from each of the *Cn*-gly-treated *Hc* displayed *Hc* with intense fluorescence staining by mAb 2D10, indicating the presence of *Cn*-gly coated *Hc* yeast (Fig. 7c). *Hc* recovered from animals challenged with *Hc* alone did not react with the GXM-binding mAb.

**Figure 8:**
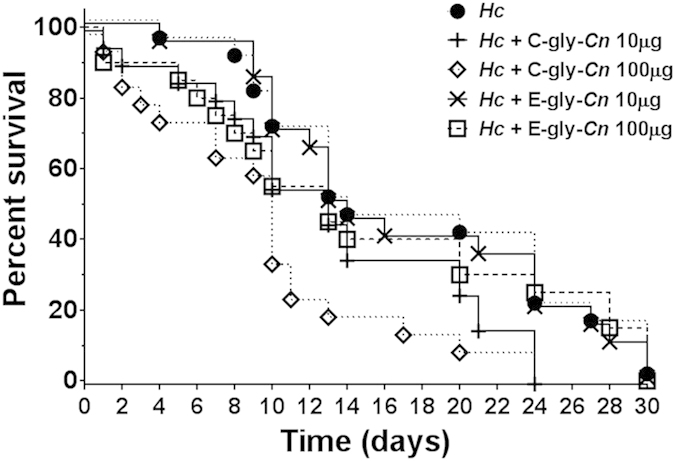
Virulence is enhanced by the incorporation of cryptococcal C-glycan fractions onto the surface of *Hc* yeast in an invertebrate infection model. Enhanced mortality was evaluated in the invertebrate *G. mellonella* model. The incorporation of C-gly-*Cn* onto the surface of *Hc* increased mortality in a dose dependent manner, with the addition of 100 μg C-gly-*Cn* producing a statistically more rapid time to death compared to untreated *Hc* (100 μg,p = 0.004; 10 μg p = 0.062). There were no significant differences between *Hc* exposed to E-gly-*Cn* coated *Hc* and *Hc* alone (100 μg, p = 0.23; 10 μg, p = 0.22).

We also tested the impact of PS-coating of *Hc* using the *in vivo* invertebrate model *Galleria mellonella* (Fig. 8); however, this approach was limited by the use of only *Hc* yeasts pre-incubated with purified *Cn*-gly. The results demonstrated a dose-dependent increase in virulence of *Hc* yeasts when coated with C-gly-*Cn* as *Hc* coated with 100 μg displayed higher virulence relative to untreated *Hc* (**p = 0.004). Treatment with 10 μg C-gly-*Cn* did not reach statistical significance compared to *Hc* alone (p = 0.062). In contrast, co-incubation with E-gly-*Cn* prior to infection resulted in similar mortality rates as that observed for the untreated *Hc* in our *Galleria* model. Nevertheless, our finding that coating of *Hc* with C-gly-*Cn* enhanced virulence in this second model strengthens our thesis that gly transfer between *Cn* and *Hc* during co-infection can enhance virulence and exacerbate disease.

### Glycan transfer occurs inside phagocytic cells

**Figure 9:**
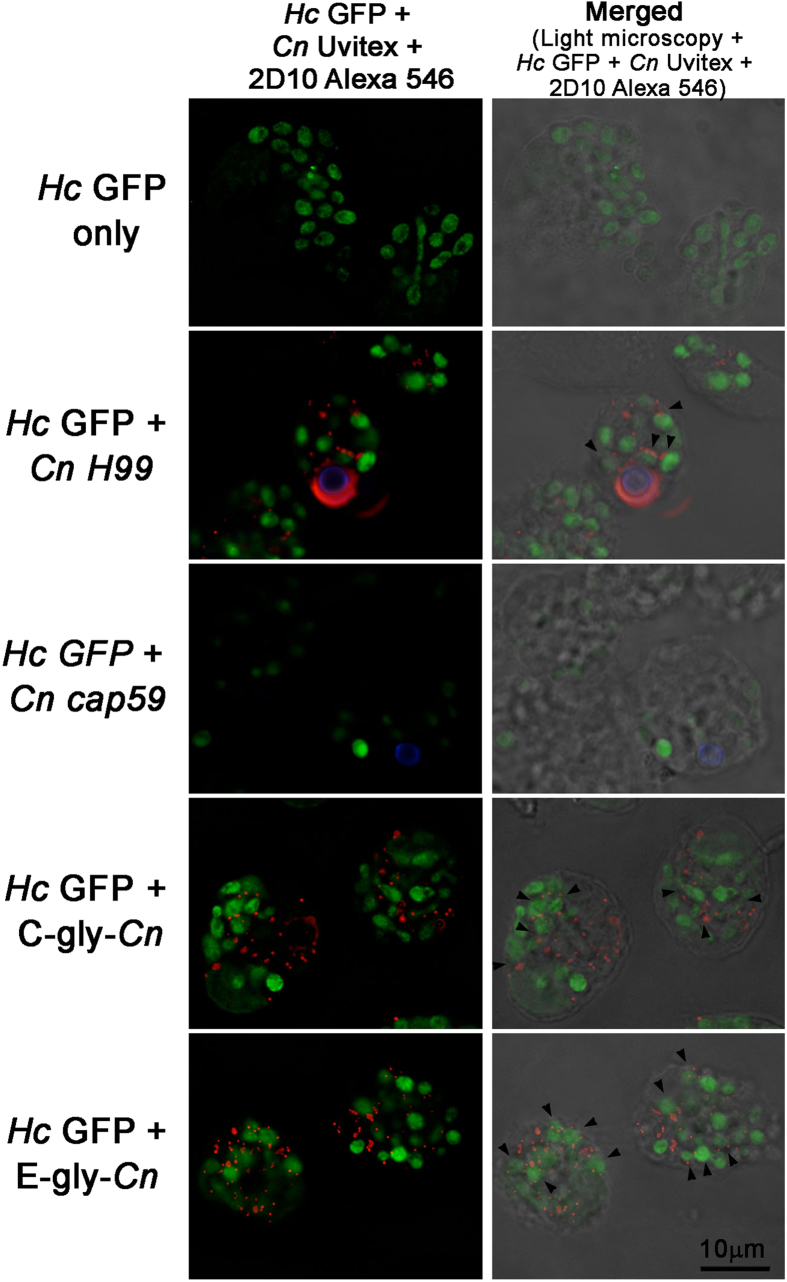
*Hc* co-localize with *Cn*-gly and is able to incorporate these glycans on its surface within the macrophage environment. Macrophages were infected with *Hc* GFP (green) and incubated with either PBS, *Cn* H99 (Uvitex labeled – blue), *Cn* cap59 (Uvitex labeled – blue), C-gly-*Cn* or E-gly-*Cn.* Fluorescence was performed using 2D10 mAb and anti-IgM Alexa 568 conjugated (red). In the presence of *Hc* GFP yeasts and either *Cn* H99, C-gly-*Cn* or E-gly-*Cn, Hc* surface was labeled with 2D10 antibody as indicated in several instances by the black arrow heads. For *Hc* and PBS or *Cn* cap59 groups, no labelling for GXM was observed. Left column (*Hc* GFP-green; *Cn* Uvitex – blue; mAb 2D10 – red). Right column (*Hc* GFP-green; *Cn* Uvitex – blue; mAb 2D10 – red) merged with light microscopy. Scale bar = 10 μm.

GXM can easily be ingested by macrophages through pinocytosis and phagocytosis^40^. *Cn* and *Hc* can also be phagocytosed by these cells and localized within phagosomes. We therefore evaluated intracellular glycan transfer within macrophage phagolysosomes. Macrophages were infected with *Hc* and then exposed to *Cn* H99, *Cn* cap59, C-gly-*Cn* or E-gly-*Cn. Hc* GFP was detected as a green fluorescent cells inside phogosomes (Fig. 9). Glycans reacting with mAb 2D10 were detected as punctuated patterns inside the macrophages in the presence of *Cn* H99, or upon incubation with C-gly-*Cn* or E-gly-*Cn* as described previosuly^40^*. Hc* GFP and *Cn* H99 co-localized within the same phagosome, and a punctuated labelling for GXM was observed around *Hc* GFP yeast (Fig. 9). When C-gly-*Cn* or E-gly-*Cn* were administered upon incubation of macrophages with *Hc,* higher distribution of GXM and labelling of *Hc* GFP yeasts by GXM antibody was observed, with a predominance of GXM staining on the surface of *Hc* yeasts (Fig. 9). Systems where *Hc* GFP was used only or where infection with *Hc* GFP was followed by *Cn* cap59 produced no labelling for GXM.

## Discussion

Histoplasmosis and cryptococcosis are the most prevalent pulmonary mycoses in HIV-infected patients^2^,^3^,^20^. *Hc var. capsulatum* infection has emerged as one of the most common systemic mycosis in the setting of HIV-infected patients in developing countries^41^, where disseminated histoplasmosis continues to cause severe morbidity and mortality. Cryptococcosis is frequently manifested in immunocompromised individuals, as meningoencephalitis particularly in the setting of HIV, with *Cn var. grubii* being the principal causative agent of the disease, followed by *Cn* var. *neoformans*^42^.

Pulmonary infections by both *Hc* and *Cn* frequently display overlapping symptoms^42^,^43^. In addition, their clinical, pathologic and imaging findings can be similar^44^. Both fungi can be isolated from bronchoalveolar aspirates, but *Cn* is able to overgrow *Hc* in culture and even inhibit its growth^22^, which may be a reflection of its simpler nutritional requirements and faster replication rate^42^. Besides culture, standard microscopic examination does not uniformly distinguish between these species, due to morphological similarity of these fungi in clinical samples, particularly when hypocapsular strains of *Cn* are involved^14^. *Cn* and *Hc* also share the ability to proliferate within macrophages and both species are considered to be facultative intracellular pathogens^10^.

A PubMed search for the words *Hc*, *Cn* and co-infection renders many hits^9^,^10^,^11^,^12^,^13^,^14^,^15^,^16^,^17^,^18^,^19^,^20^,^21^, including multi-center reports of several patients^9^,^15^,^20^, with the first co-infection observation reported by Mider *et al.* in 1947^16^. The diagnoses of co-infection was made by either histological examination and/or cultures of various tissues and body fluids. The majority of the reports date from the last decade, and are frequently associated with disseminated infection by both fungi in the setting of advanced HIV disease^9^,^12^,^13^,^15^,^17^,^18^,^20^,^21^. However, as an example of co-infection in a non-HIV infected patient, *Hc* and *Cn* were found in samples of respiratory secretions in an individual on chronic steroid therapy who presented with a cavitary pulmonary lesion^19^. In a study to validate an ELISA for the diagnosis of histoplasmosis, 12% of the histoplasmosis patients also had positive results for the presence of *Cn* by detection of GXM in the cerebrospinal fluid (unpublished and^45^). In this context, we speculate that the total number of co-infection cases is generally underestimated, primarily due to the lack of sensitivity of the methods currently in use to diagnose histoplasmosis. Additionally, the diagnosis of non-meningeal cryptococcosis is difficult^1^,^46^. However, the advent of more sensitive molecular diagnostic techniques has increased the ability for detecting *Hc* in the setting of co-infections. It should be noted that other co-infections with dimorphic fungi also occur, such as the recently reported lethal human dual infection with *Blastomyces* and *Coccidioides* spp^47^.

We postulate that interactions of *Hc* with cryptococcal GXM may contribute to the pathogenesis of a significant number of histoplasmosis cases. *Hc* and *Cn* are frequently found in the same natural sites^48^, as they are highly associated with soils enriched with organic nitrogen sources, such as animal excrements. For instance, Cermeno *et al.*^49^ co-isolated *Cn* and *Hc* from many sites in Venezuela, reinforcing the possibility of environmental interactions and an enhanced risk of co-infection with both pathogens.

In both *Hc* and *Cn*, surface PSs are key molecules of the fungal cells since they are directly mediating interactions with the immune system. *Cn* GXM is recognized by Toll-like receptors 2 and 4 and/or CD14 on phagocytes, resulting in an incomplete activation of pathways necessary for TNF-α production and activation of inflammatory responses^40^. GXM is also recognized by CD18 resulting in the blockage of the receptor, which subsequently inhibits leukocyte infiltration into inflammatory sites^23^,^40^. In *Hc*, α- and β-glucans form the outer cell wall layers of both yeast cells and mycelia, playing different biological roles^31^. The β-1, 3-glucan, which predominates in the mycelial phase, is antigenic and modulates the host immune response^31^. In most *Hc* isolates, α-1, 3-glucan surrounds the β-1, 3-glucan layer, blocking its innate recognition by dectin-1 on host phagocytes^50^, and thereby suppressing the production of TNF-α^31^.

The interaction between *Cn* and *Hc* can result in hybrid pellicle formation. We found that pellicle formation was increased when GXM producing *Cn* yeast cells were co-incubated with *Hc*. This observation suggests that cell wall components of *Hc* could interact with *Cn-*gly to promote adhesion of matrix components resulting in effective pellicle formation. In fact, *Hc* can incorporate exogenous *Cn* GXM but the mechanism by which PSs are attached to *Hc* cell surfaces remains obscure. Previous reports have demonstrated that only an α-1, 3-glucan-producing *Hc* strain could anchor soluble GXM based on immunofluorescence staining^25^. However, no direct labelling control of mAbs to *Hc* was performed. In our system, strain G217B, which displays no α-1, 3-glucans, had a slightly lower efficacy in incorporating C-gly-*Cn* in comparison with the strain G186A, a well-recognized α-1, 3-glucan-producing strains^39^, possibly indicating that *Hc* α-1, 3-glucans are not specific determinants for interaction with cryptococcal gly. The PS adsorption was more efficient when C-gly*-Cn* were used in comparison with E-gly-*Cn*. Coating *Hc* with C-gly-*Cn* also resulted in an increase in the magnitude of the fungal cell’s negative charges, most likely due to the fact that this cellular fraction was better incorporated onto the cell surface and that it has higher amounts of glucuronic acid residues than the extracellular soluble fraction, E-gly-Cn^38^.

In addition, the incorporation of *Cn*-gly by *Hc* in both environmental and infection-related conditions, may have the potential to modify the outcome of the interaction between yeasts and phagocytes and/or environmental predators. Such an altered outcome was observed with the environmental *in vivo* model *G. mellonela*, which likely favors the survival of both microorganisms under environmental stress conditions and/or during interactions with the innate immune system. Coating of *Hc* with crypotoccocal PS might also inhibit the interaction with phagocytes, including macrophages, dendritic cells, neutrophils in mammalian models and haemocytes in *G. mellonella* invertebrate model. Within phagocytic cells, as shown with macrophages, GXM is extensively released by *Cn* in the phagosome^51^. In the case of co-infection of a single magrophage, as shown, the *Cn*-gly could be incorporate and associate with *Hc* yeast cell surface.

The results presented in this study suggest that *Cn* and *Hc* share a number of physiological steps required for gly formation and surface assembly. In addition, they also reveal a new pathogenic mechanism, resulting in increased virulence or synergism, with potential relevance for hosts co-infected with these fungi. Our *in vivo* observations suggest that these fungal pathogens can interact during infection, and *Hc* could modify its cell surfaces in a manner that alters recognition by the immune system. The explanation for the *Cn*-gly incorporation effect on *Hc* virulence may primarily be due to a subversion of the host immune recognition mechanisms of cell wall components with subsequent increase in yeast survival, which is an effect observed when comparing highly capsulated *Cn* strains to minimally capsulated ones^30^. Hence, direct PS transfer resulted in enhanced *Hc* virulence associated with the suppression of the antifungal functions of phagocytic cells.

Our findings also suggest that an increased understanding of the role of PS in fungal infections may lead to promising strategies for the design of new therapeutics^37^,^52^, as PSs constitute important targets for vaccines and passive immunization^53^. The mechanism used by fungal cells to incorporate exogenous molecules with consequent change of their surface architecture consists of a new avenue for cell biology studies and likely for the design of new therapeutic options. In summary, our findings show that *Hc* can co-opt GXM, the major virulence factor of *Cn*, during mixed infection *in vivo* and that this phenomenon was associated with increased virulence, both *in vitro* and *in vivo*. This observation establishes the precedent of one pathogenic microbe using a virulence factor from another to increase its virulence, suggesting that other such interactions may exist in host-microbe relationships. Although this is a new concept for synergistic dual fungal infection, the paradigm is well known in bacterial diseases and increasingly emerging in fungal-bacterial infections. For example, infection with mixed bacterial species can produce synergisms in virulence resulting in severe disease, such as Fournier gangrene. Bacterial-fungal interactions such as those described for *Pseudomonas aeruginosa* and *Candida albicans* can affect the expression of several fungal characteristics including some associated with virulence^54^. Our experiments extend the phenomenon of microbial synergy in virulence due to mixed infections within the fungal kingdom.

## Methods

### Fungal strains and growth conditions

*Cn* var. *grubii* Serotype A strain H99 (ATCC 208821), the acapsular mutant *Cn cap59* (derivative of Serotype D strain B3501 ATCC 34873), *Hc* var. *capsulatum* G217B, *Hc* G217B GFP (kind gift from Dr. A. G. Smulian, Division of Infectious Diseases, University of Cincinnati College of Medicine, Cincinnati, Ohio, USA) and *Hc* G186A (ATCC 26029) were used in this study. *Cn* was cultured in minimal media (29.4 mM KH_2_PO_4_, 10 mM MgSO_4_, 13 mM Glycine, 3 μM Thiamine and 15 mM D-Glucose, pH 5.5). *Hc* was cultured in HAM’s F-12 (Invitrogen) medium as described^55^. *Cn* and *Hc* cells were grown at 30 °C and 37 °C, respectively, for 48 h with shaking at 150 rpm. For the co-cultivation of both fungi, *Hc* and *Cn* were centrifuged at 1100 × g for 10 min at room temperature (RT) and pellets were washed three times with PBS followed by centrifugation. The cells were then suspended in HAM’s F-12 and enumerated using a hemocytometer. *Hc* and *Cn* yeasts were added to a final density of 5 × 10^5^ yeasts/mL in HAM’s F-12 and co-cultures were incubated at 30 °C and 37 °C. Monospecies controls of *Hc* (G217B of GFP) or *Cn* (H99) at 10^6^ yeasts/mL were incubated separately in 50 mL of HAM’s F-12 at 37 °C and 30 °C.

### Mouse co-infection model

To evaluate survival rates during co-infection *in vivo*, C57BL/6 mice (6–8 weeks old) were challenged intranasally with 5 × 10^6^ *Hc* GFP, followed 2 h later by an intratracheal infection with 5 × 10^6^ of *Hc* GFP (monospecies control), *Cn* H99 or *Cn* cap59. The infected mice were checked four times daily by the scientific team and daily by the veterinary staff. All animal experiments were carried out in “accordance” with the approved guidelines and protocols of the Institutes for Animal Studies at the Albert Einstein College of Medicine and the Fluminense Federal University. To determine fungal burdens, immediately after they were detected, deceased animals had their lungs removed and the organs were then weighed and homogenized in PBS using 70 μm cell strainers (BD Biosciences, NJ, USA). Organ homogenates were serially diluted and plated in duplicates on Sabouraud dextrose agar (Difco Laboratories) for *Cn* growth. After 2 d of incubation at 30 °C, *Cn* colony forming units (CFUs) were enumerated. For *Hc* growth determination, homogenates were simultaneously also plated on brain heart infusion (BHI) agar supplemented with 5% sheep blood and bleomycin at 10 μg/mL (to suppress *Cn* growth in co-infection conditions). BHI plates were incubated in the dark for 10–15 d at 37 °C and *Hc* CFUs were then enumerated. The plates were also observed under UV light for expression of GFP proteins by *Hc* GFP strain and correlated with colony morphology.

To examine cryptococcal gly incorporation by *Hc* during co-infection, aliquots of lung homogenates were spun down and evaluated by immunofluorescence. Homogenates were pipetted into microcentrifuge tubes and quickly spun down to remove excess liquid. For detecting bound *Cn* PS, *Hc* yeasts were incubated with 10 μg/mL of the IgM isotype GXM-binding mAb 2D10 or isotype-matched irrelevant antibody^33^ and a 1:100 of a goat anti-mouse IgM Alexa 546 conjugate. After three washes, fungi were stained using 0.5 mg/mL of Uvitex 2B, fixed with 4% paraformaldehyde and analysed in an AX70 fluorescence microscope. Alternatively, we used goat anti-mouse IgM APC conjugate and performed analysis of FL1+FL4+cells (GFP and APC labelled, respectively) using a FACScalibur Flow Cytometer (BD Biosciences, Franklin Lakes, NJ) and *Hc* fluorescence intensity was determined under each condition.

### *Hc* pellicle formation induced by *Cn* or their products

Monospecies cultures of *Hc* and *Cn* yeasts were obtained as described above, collected by centrifugation, washed with PBS (3X), and suspended at 10^7^ cells/mL in HAM F-12 media. An aliquot of *Hc* yeast suspension was heat-killed at 56 °C for 1 h and used as negative control. Next, 100 μL (10^6^ total yeast) of each suspension (*Hc* or *Cn*) was added to individual wells of polystyrene 96-well plates (Fisher, MA). In co-incubations conditions, 50 μL (5 × 10^5^) of *Hc* GFP and 50 μL (5 × 10^5^) of *Cn* H99 or *Cn* cap59 were added to the same well (10^6^ total yeast cells per well). Plates were incubated at 37 °C without shaking for 48 h. Following incubation, wells were washed (3X) with PBS 0.05% Tween 20 to remove planktonic cells. Pellicle formation, as agglutination of cells on a surface, was measured by XTT (2, 3-bis (2-methoxy-4-nitro-5-sulfophenyl)-5-[(phenylamino) carbonyl]-2*H*-tetrazolium-hydroxide) reduction assay as previously described^34^. Briefly, 50 μL of XTT salt solution (1 mg/mL) in PBS and 4 μL of menadione solution (1 mM in acetone) were added to each well and plates were incubated for 5 h at 37 °C. Changes in color and reduction of XTT tetrazolium salt into XTT formazan by fungal mitochondrial dehydrogenase correlate with metabolic activity and cell viability. The absorbances were measured at 492 nm using a microplatereader (SpectraMax Microplate Reader, Molecular Devices, CA, USA). The conditions were tested in quadruplicates and the results shown are the average of three independent experiments. The background activity of heat-killed *Hc* was discounted from all the wells as a blank control. A similar plate set-up was then used for visual documentation of the pellicle architecture by immunofluorescence of *Hc*-GFP yeast^34^.

To determine the pellicle formation and initial accumulation of PS extracellular matrix component, an ELISA with IgM mAb 2D10 was performed^33^. After 48 h incubation, plates were washed (3X) with TBS-T (10 mM Tris-HCl, 150 mM NaCl, 1 mM NaN_3_, 0.1% Tween 20, pH 7.4) and incubated with blocking solution (2% Bovine Serum Albumin in TBS-T) for 1 h at 37 °C. After washes, mAb 2D10 was diluted at 10 μg/mL in blocking solution. Fifty microliters of mAb solution was added to separate wells containing yeast cells in quadruplicate and the plates were incubated at 37 °C for 1h. An irrelevant IgM antibody 5C11 was used as a control^56^. Plates were washed (3X) with TBS-T and incubated with a 1:1000 dilution of a goat anti-mouse Ig (Southern Biotech) in blocking solution, for 1h at 37 °C. After washes (3X), plates were incubated with 50 μL/well of 1 mg/mL p-nitrophenyl phosphate diluted in substrate buffer (1 mM MgCl_2_ × 6H_2_O, 0.05 M Na_2_CO_3_, pH 9.8) at 25 °C for 30 min. Absorbances were measured at 405 nm on a microplate reader (BioTek Instruments, Winooski, Vermont, USA). Results shown are the average of 3 independent experiments.

### Analysis of cross-incorporation of *Cn* polysaccharides

Co-cultivated *Hc* GFP and *Cn* yeasts at different temperatures (30 and 37 °C) were washed (3X) with PBS and incubated with 10 μg/mL of mAb 2D10 or irrelevant isotype-matched antibody, diluted in blocking solution for 1 h at RT. As a control, *Hc* and *Cn* were mixed right before incubation with mAb. The yeasts were washed and suspended in 100 μL of a goat anti-mouse IgM APC-conjugate (Southern Biotech) diluted 1:100 in blocking solution. The suspension was incubated for 1h at RT and washed with PBS. Cells were sonicated with 1 min cycles to disrupt any possible aggregates or biofilm formed during growth/incubations, fixed for 20 min using formalin buffer (Fisher Scientifics) and washed with excess of PBS. Analysis of FL1+FL4+cells (GFP and APC double-labelled) was performed in a FACScan Flow Cytometer (BD Biosciences, Franklin Lakes, NJ) and fluorescence intensity was determined for each condition.

Alternatively, *Hc* grown in the filamentous phase on microslides at RT were incubated with 100 μg/mL of total PS obtained from *Cn* culture supernatants^25^,^38^ for 1h at RT. Similarly, *Hc* yeast were adhered to poly-L-lysine coated slides and incubated with *Cn* PS. Slides were then washed and incubated with 10 μg/mL of mAb 18B7^37^ to GXM or isotype-matched antibody and a 1:100 of a goat anti-mouse FITC-conjugated Ab. As a control for glycan incorporation through the requirement of cell surface carbohydrates or proteins, cells were treated with Novozyme 234 (Novoenzyme, Windsor, UK), a multi-enzyme preparation containing carbohydrate and peptide hydrolases^57^. After three washes, fungi were stained using 0.5 mg/mL of Uvitex 2B and fixed with 4% paraformaldehyde. Glycan incorporation was examined with an immunofluorescence Olympus AX70 fluorescence microscope, with a magnification of 40X.

### Isolation of fungal glycans

Two-day old 1 L cultures of *Cn* H99 yeasts were centrifuged for 10 min at 1100 × *g*. Both cells and culture supernatants were collected for the extraction of cellular attached gly (C-gly) and isolation of secreted extracellular gly (E-gly), respectively. C-gly extraction was performed with DMSO as described^38^. E-gly were obtained by ultrafiltration of the supernatant using nitrocellulose membranes with a nominal molecular weight limit (NMWL) of 10 kDa (Millipore, MA, USA) as described^38^. Concentrated E-gly and C-gly were dialyzed against water for 24 h (with at least 8 water exchanges) and then lyophilized. The *Cn*-gly were quantitated by inhibition ELISA as described^58^.

### Incorporation of cryptococcal cellular and extracellular glycan fractions by *Hc*

C-gly-*Cn* and E-gly-*Cn* (100 μg) were incubated with 10^7^ GFP *Hc* yeasts for 1 h at 37 °C in PBS. *Hc* yeasts incubated in PBS alone were used as a control. Following incubation, cells were washed (3X) with PBS to remove unbound gly and enumerated using a haemocytometer. Gly incorporation by *Hc* GFP yeast was determined by FACS analysis using mAb 2D10 as described above.

*Hc* were also suspended at 10^7^ cells/mL in HAM F-12 medium. Next, 50 μL (10^6^ total yeast) was added to individual wells of polystyrene 96-well plates (Fisher, MA) and incubated with 10μg of C-gly-*Cn* or E-gly-*Cn* in 50 μL of HAM F-12 media). Plates were incubated at 37 °C without shaking for 48 h. Pellicle formation was assessed as described previously.

To compare the relative incorporation of *Hc* G217B with the high α-1, 3-glucan strain *Hc* G186A, yeasts were incubated with the *Cn* gly fractions for different time intervals and incorporation was detected by indirect ELISA as described^58^.

### Zeta potential measurements

*Hc* zeta potential was examined before and after incubation with C-gly-*Cn* or E-gly-*Cn*, and included untreated *Hc* yeast as a control. Analysis was done using 10^6^ cells/mL in pure distilled LPS free water (Thermo Scientific HyClone). Zeta potential (ζ) and mobility values of intact cells were measured in a Zeta potential analyser (ZetaPlus, Brookhaven Instruments Corp., Holtsville, NY) as described^58^.

### Scanning electron microscopy

Acapsular *Cn* cap59 mutant, *Hc* or the *Cn*-gly-coated yeasts were washed three times in PBS and fixed with 0.1 M sodium cacodylate buffer containing 2.5% glutaraldehyde for 1 h. Yeast were washed with 0.1 M sodium cacodylate, 0.2 M sucrose and 2 mM MgCl_2_ and fixed on coverslips coated with poly-L-lysine for 20 min. Preparations were then gradually dehydrated in alcohol (30%, 50%, 70% and 100% for 5 min and 95% and twice in 100% for 10 min), and submitted to critical point drying and metallization. The cells were observed in a Quanta-FEI scanning electron microscope (FEI,USA).

### Phagocytosis

Four-to-six weeks-old female BALB/c mice were used for the isolation of peritoneal macrophages^59^. Macrophages were plated onto a culture chamber at 2 × 10^5^ cells/well. *Hc* yeasts were labelled with 40 μg/mL of NHS Rhodamine (Thermo Scientific, Rockford, lL, USA) for 30 min at 25 °C and washed (3X) with excess of PBS. Cells were incubated with the distinct *Cn*-gly or PBS as described above. Following incubation, cells were washed, suspended in DMEM, enumerated, and added to the macrophages in a 5:1 (yeast:macrophage) ratio. Plates were incubated for 1 h in 5% CO_2_ atmosphere. After three washes with PBS, yeasts were stained using 0.5 mg/mL of Uvitex 2B to distinguish internalized versus extracellular yeasts. Wells were washed (3X) with PBS and fixed with a 4% formaldehyde solution in PBS. The number of macrophages and yeasts were recorded for each field by microscopic enumeration and at least 200 macrophages were counted. The percentage of phagocytosis was determined as the ratio of macrophages with internalized yeast cells divided by total macrophages, and the phagocytic index as the average number of yeast inside macrophages^55^.

### Yeast killing assay

*Cn*-gly-coated *Hc* yeast cells were suspended in DMEM and added in a 5:1 (yeast:macrophage) ratio to 96-well culture plates containing 10^5^ macrophages/well. Plates were incubated overnight at 37 °C under 5% CO_2_. The wells were washed with cold PBS and macrophages lysed by adding sterile water. Aliquots were plated onto BHI-blood agar plates (10 g/L glucose, 0.1 g/L cysteine, 1% Pen-Strep and 5% v/v sheep red blood cells) and incubated at 37 °C for 10–15 days. The numbers of CFUs were enumerated and compared among groups.

### Nitric oxide synthase activity

Nitric oxide production by peritoneal macrophages following incubation with control or gly-coated *Hc* yeast cells was determined from culture supernatant using the Griess reagent (Promega, Madison, WI, USA) according to manufacturer’s instructions. A nitrite standard reference curve was prepared for accurate quantization of NO_2_ levels in experimental samples. Experimental conditions were performed in quadruplicates. Plates were read in a spectrophotometer at 540 nm.

### Survival in mammalian and invertebrate host models against *Cn*-PS coated *Hc*

Mice were intranasally infected with 10^7^ *Hc* GFP yeast followed 2h later by intratracheal injection with 10 μg (in 50 μL) of C-gly-*Cn*, E-gly-*Cn* or PBS. Mice were checked four times daily by the scientific team and daily by the veterinary staff. Evaluations of *Hc* virulence were performed by survival and CFU quantification as described previously.

To further assess the effects of the incorporation of distinct gly pools by *Hc* in pathogenesis, survival experiments were also conducted in *Galleria mellonella* according to our established methods^60^. Prior to infection, *Hc* yeast cells were treated with cellular C-gly-*Cn*, extracellular E-gly-*Cn* or PBS (control) as described above. Infections were performed by injecting the hemocoel of each caterpillar via the last left proleg with 10μL aliquot containing 10^6^ yeast using a 10-μl Hamilton syringe. Groups consisted of 10 larvae per group and experiments was repeated 3 times with similar results achieved.

### Model of glycan transfer during infection of macrophages

Peritoneal macrophages (2 × 10^5^ in 200 μL) were plated on 8-chambers culture slides (Falcon) and cultivated overnight at 37 °C under 5% CO_2_. *Hc* GFP yeasts were washed and added to macrophages at a 2:1 ratio, and infection performed for 2 hours. Chambers were washed three times with DMEM to remove extracellular *Hc* GFP yeasts. Cn yeasts were incubated with Uvitex 2B as described above, and either *Cn* H99 or *Cn* cap59 were added to macrophages in a 5:1 ratio. For C-gly-*Cn* or E-gly-*Cn*, glycans were diluted at 10 μg/mL in 200 μL of DMEM and added to individual wells. Chambers were incubated overnight at at 37 °C under 5% CO_2_. After washing with PBS, chambers were fixed as described and immunofluorescence conducted as described above.

### Statistical analysis

All analyses were performed using GraphPad Prism version 6.00 for Windows (GraphPad Software, San Diego California USA). One-way ANOVA statistics using a Kruskall-Wallis non-parametric test was used to compare the differences among groups with a 95% confidence interval in all experiments. Individual comparison between groups was performed using Bonferoni post-test. Survival results were analyzed by Kaplan-Meyer to determine the difference among groups.

## Additional Information

**How to cite this article**: Cordero, R. J. B. *et al.* Enhanced virulence of *Histoplasma capsulatum* through transfer and surface incorporation of glycans from *Cryptococcus neoformans* during co-infection. *Sci. Rep.* **6**, 21765; doi: 10.1038/srep21765 (2016).

## Supplementary Material

Supplementary Information
