# Supplementary material for: Enhanced virulence of Histoplasma capsulatum through transfer and surface incorporation of glycans from Cryptococcus neoformans during co-infection
Source: Sci Rep. 2016 Feb 24;6:21765. doi: 10.1038/srep21765 (PMC4764860; doi:10.1038/srep21765)
Supplement: Supplementary Information [file srep21765-s1.pdf]

Enhanced virulence of *Histoplasma capsulatum* through transfer and surface incorporation of glycans from *Cryptococcus neoformans* during co-infection

Radames J. B. Cordero, Susie Coutinho Liedke, Glauber de Souza Araújo, Luis R. Martinez, Leonardo Nimrichter, Susana Frases, Jose Mauro Peralta, Arturo Casadevall, Marcio L. Rodrigues, Joshua D. Nosanchuk and Allan Jefferson Guimaraes

SUPPLEMENTARY MATERIAL:

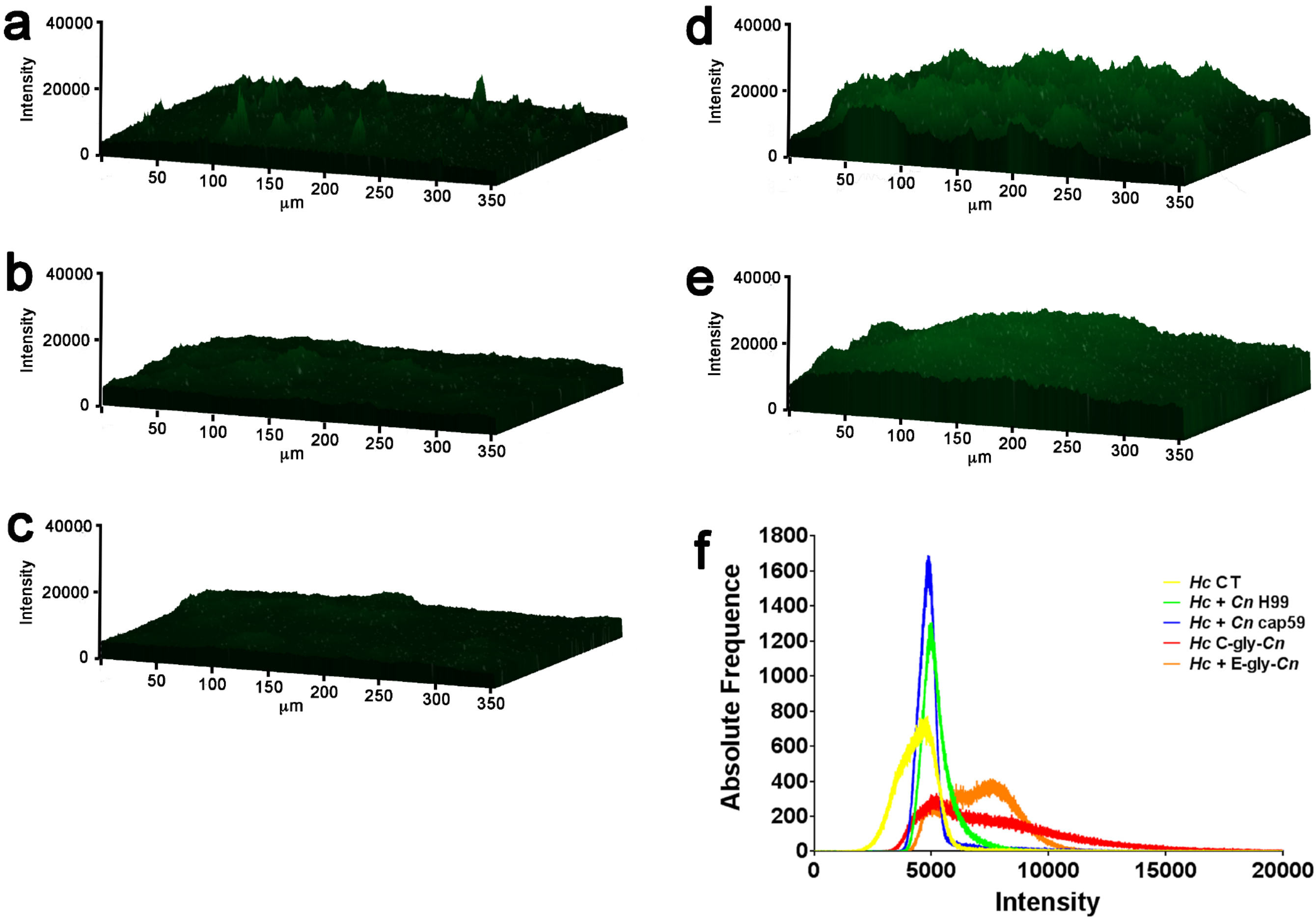

Supplementary Figure 1. Structural determination of pellicles by a 3D-rendering model obtained by Z-stack imaging and deconvolution using Zeiss software. (a) *Hc* GFP; (b) *Hc* GFP + *Cn* H99 (c) *Hc* GFP + *Cn* cap59 (d) *Hc* GFP + C-gly-*Cn* (e) *Hc* GFP + E-gly-*Cn.* (f) Plot displays the frequency of fluorescent areas after imaging analysis and an increase was observed for *Hc* GFP + *Cn* H99, *Hc* GFP + C-gly-*Cn* and *Hc* GFP + E-gly-*C,* displaying the importance of Cn-gly on *Hc* floculation. *Hc* CT indicates *Hc* alone.

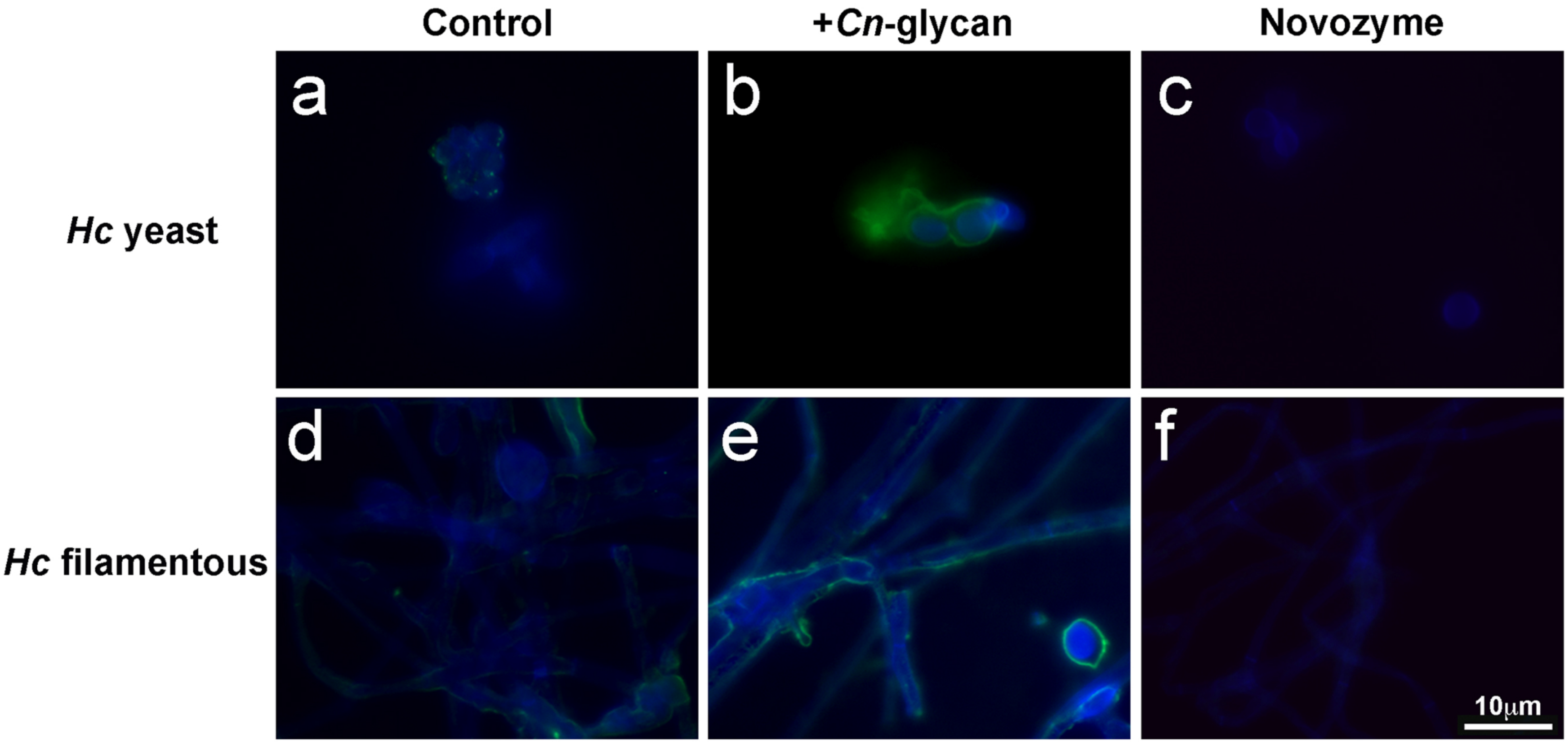

Supplementary Figure 2. *Cn*-glycan incorporation occurs in both the filamentous and yeast phase of *Hc*. Micrograph representations of *Hc* yeast cells (upper row: a, b and c) and hyphae (lower row: d, e and f) labeled with GXM-binding mAb 18B7 and an anti-IgG Alexa 488 conjugate (green) before (a and d) and after (b and e) incubation with *Cn*-glycan. (c and f) Binding of *Cn*-gly to the *Hc* surface was prevented when cells were pre-incubated with cell wall digesting enzymes (Novozyme). Cells were also labelled with Uvitex (blue) to denote the limits of the fungal cell wall. Scale bar = 10μm.

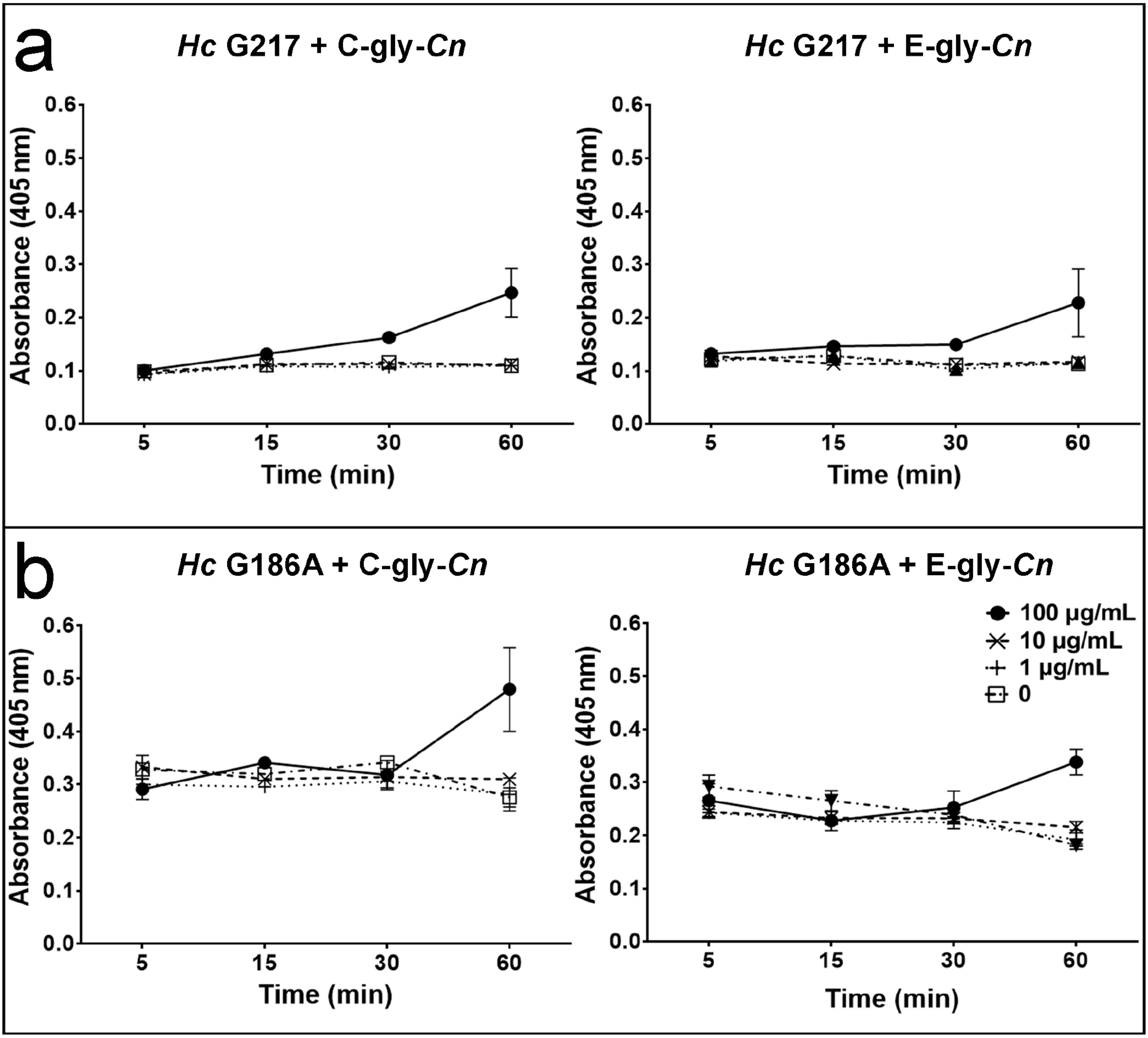

Supplementary Figure 3. Kinetics of cryptococcal glycan incorporation differed among distinct *Hc* strains with different amounts of α-1,3-glucans on their surface: (**a**) G217B, low α-1,3-glucan producing and (**b**) G186A high α-1,3-glucan producing strain. Statistically significant incorporation was observed at incubations longer than 30 min for strain G217B for both C-gly-*Cn* or E-gly-*C,* while for G186 strain, difference was observed at 60min for both GXM fractions (*p<0.05).
